# Psychometric Properties of Questionnaires on Functional Health Status in Oropharyngeal Dysphagia: A Systematic Literature Review

**DOI:** 10.1155/2014/458678

**Published:** 2014-04-29

**Authors:** Renée Speyer, Reinie Cordier, Berit Kertscher, Bas J Heijnen

**Affiliations:** ^1^School of Public Health, Tropical Medicine and Rehabilitation Sciences, James Cook University, Townsville, QLD 4811, Australia; ^2^Department of Otorhinolaryngology and Head and Neck Surgery, Leiden University Medical Center, 2333 ZA Leiden, The Netherlands; ^3^School of Occupational Therapy and Social Work, Curtin University, Perth, WA 6845, Australia; ^4^RehaA Wil and RehaA Winterthur, RehaClinic, 5330 Bad Zurzach, Switzerland

## Abstract

*Introduction*. Questionnaires on Functional Health Status (FHS) are part of the assessment of oropharyngeal dysphagia. *Objective*. To conduct a systematic review of the literature on the psychometric properties of English-language FHS questionnaires in adults with oropharyngeal dysphagia. *Methods*. A systematic search was performed using the electronic databases Pubmed and Embase. The psychometric properties of the questionnaires were determined based on the COSMIN taxonomy of measurement properties and definitions for health-related patient-reported outcomes and the COSMIN checklist using preset psychometric criteria. *Results*. Three questionnaires were included: the Eating Assessment Tool (EAT-10), the Swallowing Outcome after Laryngectomy (SOAL), and the Self-report Symptom Inventory. The Sydney Swallow Questionnaire (SSQ) proved to be identical to the Modified Self-report Symptom Inventory. All FHS questionnaires obtained poor overall methodological quality scores for most measurement properties. *Conclusions*. The retrieved FHS questionnaires need psychometric reevaluation; if the overall methodological quality shows satisfactory improvement on most measurement properties, the use of the questionnaires in daily clinic and research can be justified. However, in case of insufficient validity and/or reliability scores, new FHS questionnaires need to be developed using and reporting on preestablished psychometric criteria as recommended in literature.

## 1. Introduction

Oropharyngeal dysphagia is associated with high mortality rates [1]. Dysphagia can lead to increased risk of dehydration, malnutrition, aspiration pneumonia, and death. Oropharyngeal dysphagia may also have a major impact on a patient's health-related quality of life and well-being [2–4]. Early detection through screening is an essential first step in the management of dysphagia [5, 6]. After being identified as being at risk of having dysphagia, further assessment of the swallowing function is required. Videofluoroscopy (VFS) and fiberoptic endoscopic evaluation of swallowing (FEES) are mooted in literature to be the gold standards in the assessment of dysphagia. Another important step after screening is the completion of patient self-administered questionnaires. Such inventories are designed to measure either health-related quality of life (HR-QoL) or functional health status (FHS) [6]. HR-QoL refers to the unique personal perception individuals may have of their health, taking into account social, functional, and psychological issues, whereas FHS is the influence of a given disease on particular functional aspects [7]. Within the context of oropharyngeal dysphagia assessment, FHS questionnaires aim to quantify the symptomatic severity of dysphagia as experienced by the patient.

The use of a particular tool to evaluate a patient's current health status or the effects of a medical intervention, whether for screening or assessment of oropharyngeal dysphagia, can only be justified if it has demonstrated reliability and validity. Systematic literature reviews have been published on the psychometric properties of oropharyngeal dysphagia screening [8, 9] and HR-QoL questionnaires [10] but not on FHS questionnaires.

The purpose of this systematic literature review is to (a) provide an overview of existing FHS questionnaires, (b) determine the corresponding psychometric properties, and (c) provide recommendations for the use of FHS questionnaires in both clinical practice and in research.

## 2. Methods

A systematic literature search was performed by two independent reviewers using two electronic databases: Pubmed and Embase. All appropriate journal articles up to June 2013 were included. To ensure that a comprehensive approach was adopted to retrieve relevant publications, mesh and thesaurus terms were supplemented by free text words (see Table 1). Only original research articles describing FHS questionnaires in oropharyngeal dysphagia were included. The search was limited to publications and questionnaires written in English. Reviews, case reports, and editorials were excluded, as well as questionnaires not related to oropharyngeal dysphagia (e.g., esophageal dysphagia or gastroesophageal reflux disease) or questionnaires mainly focusing on health-related quality of life (HR-QoL), generic questionnaires, or questionnaires targeted at population groups other than adults with oropharyngeal dysphagia (e.g., children or health care providers). Reference lists of all included articles were searched for additional literature. Differences of opinion about the inclusion of articles were settled by group discussion and reaching consensus.

Next, an extended search was conducted for each of the included questionnaires to ensure that all articles on their development and validation were retrieved using the names of each questionnaire in combination with their respective acronyms. The psychometric properties of the included questionnaires were determined using the COSMIN taxonomy of measurement properties and definitions for health-related patient-reported outcomes [11] (see Table 2). The COSMIN checklist [12] was used as a standardised tool to evaluate the methodological quality of studies on psychometric properties. The COSMIN checklist consists of nine domains, each dealing with one of the following psychometric properties: internal consistency, reliability (relative measures: including test-retest reliability, interrater reliability, and intrarater reliability), measurement error (absolute measures), content validity (including face validity), structural validity, hypotheses testing, cross-cultural validity, criterion validity, and responsiveness. Interpretability is not considered to be a psychometric property. Each domain of the COSMIN checklist contains 5 to 18 items on aspects of study design and statistical methods. The methodological quality scores per psychometric property were calculated using a 4-point rating scale according to Terwee et al. [13]: excellent, good, fair, and poor. An overall methodological quality score per psychometric property is obtained by taking the lowest rating of any item in the corresponding domain. Psychometric ratings were discussed and agreed upon during consensus meetings. If applicable, evidence from different studies on the psychometric properties of the same questionnaire was summarised by combining the results as proposed by the Cochrane Back Review Group [14].

## 3. Results 

### 3.1. Systematic Literature Search

The findings of the literature search using both Pubmed and Embase resulted in a total of 2,703 abstracts. Twelve original questionnaires were identified (see Table 3). Of those, two questionnaires were excluded because they contained mainly items on health-related quality of life: the Dysphagia Handicap Index by Silbergleit et al. [15] and the MD Anderson Dysphagia Inventory by Chen et al. [16]. Four questionnaires were excluded because they were developed in a language other than English: the French Deglutition Handicap index [17], the Dysphagia Short Questionnaire [18], the Dysphagia in Multiple Sclerosis questionnaire [19], and the Swallowing Disturbance Questionnaire [20] were developed in French, Swedish, Italian, and Hebrew, respectively. The target population of the Mayo Dysphagia Questionnaire-30 [21] consisted of patients with reflux related disorders and was therefore excluded. Similarly the Dysphagia Disorders Survey [22], a questionnaire used by speech pathologists during mealtime observation of residential populations with intellectual disabilities, was excluded as well as the Caregiver Mealtime and Dysphagia Questionnaire [23], a questionnaire that focused on caregiver compliance.

Finally, three self-administered questionnaires were included: the Eating Assessment Tool (EAT-10) [24], the Swallowing Outcome after Laryngectomy (SOAL) [25], and the Self-report Symptom Inventory [26]. The Sydney Swallow Questionnaire (SSQ) [27] is identical to the previously published Self-report Symptom Inventory by Wallace et al. [26]. All three questionnaires represent original English-language FHS Questionnaires for adult patients with oropharyngeal dysphagia.

### 3.2. Functional Health Status Questionnaires

Table 4 provides information on the development of the EAT-10, the SOAL, and the Self-report Symptom Inventory. Initially, a Prototype Self-report Symptom Inventory was developed by Wallace et al. [26]. During the validation and reliability process of this prototype, the final version or the Modified Self-report Symptom Inventory was created. As the SSQ [27] is identical to the Modified Self-report Symptom Inventory, the SSQ is subsumed under the Modified Self-report Symptom Inventory.  Table 5 gives an overview of the studies that were involved in the validation of the questionnaires. Both Tables 4 and 5 list the questionnaires included, the developmental and/or validation studies, the applied study designs, the study populations involved, and the subject characteristics of the target population.

Finally, Table 6 includes the characteristics of all three FHS questionnaires. All questionnaires contain one domain with the exception of the SSQ. Although the Modified Self-report Symptom Inventory is identical to the SSQ, Dwivedi et al. [27] distinguish the domain of physiological swallow function from two separate items: one item on overall swallowing function and another on swallowing-related quality of life (HR-QoL). Upon closer inspection, the items of the other questionnaires also included similar questions on HR-QoL. For example, the EAT-10 items, “The pleasure of eating is affected by my swallowing” and “Swallowing is stressful” could be considered to be HR-QoL questions rather than FHS questions. A similar observation could be made in the case of the SOAL-item, “Has your enjoyment of food reduced?.” However, as the majority of items of all the questionnaires focus on FHS, the influence of a few HR-QoL items was considered to be unimportant.

The number of items varies between 10 and 19 items per questionnaire. The EAT-10 includes ten items using 5-point Likert scales (from “no problem” to “severe problem”), whereas the SOAL consists of 17 items using three response options: “no,” “a little,” or “a lot.” Both the Self-report Symptom Inventory and the SSQ consist of mainly visual analogue scales. The lowest score for all questionnaires is zero (last impaired), whereas the highest possible scores range between 34 (SOAL) and 1708 (Prototype Self-report Symptom Inventory).

### 3.3. Psychometric Properties

The psychometric properties of all three FHS questionnaires were examined using the COSMIN taxonomy of measurement properties and definitions for health-related patient-reported outcomes [11]. Using the COSMIN checklist [12] and the 4-point rating scale according to Terwee et al. [13], overall scores of methodological quality for each measurement domain were obtained. The cross-cultural validity domain was not evaluated as only original English-language questionnaires were included in the systematic literature review. The summarised psychometric consensus ratings of all questionnaires are depicted in Table 7. All statements on the rating of the methodological quality per measurement domain of each questionnaire in the next few paragraphs refer to the “worse score counts” criteria as described by Terwee et al. [13].

#### 3.3.1. EAT-10 [24]

No factor analysis was performed to determine* internal consistency*. As Belafsky et al. [24] are the first to report on the EAT-10, no reference was provided to another study that would provide this information.* Reliability* scored poorly as no weighted or unweighted Kappa and no percentage agreement information were reported. Pearson product moment correlations were calculated instead of ICCs. Therefore, the authors did not account for possible systematic differences in their data. Because no Standard Error of Measurement (SEM) was determined,* measurement error* scored poorly. No reference to age, gender, disease characteristics, country, or setting was considered during item selection. No evaluation was conducted to determine if all ten items reflected the construct (dysphagia). As a result,* content validity* scored poorly. No information was reported on* structural validity*. No information was provided on describing the constructs or measurement properties of the comparator instruments resulting in a poor rating on* hypotheses testing*.* Criterion validity* was not assessed. In relation to* responsiveness,* no information was provided on the description of the constructs or measurement properties of the comparator instruments. The criterion used could not be considered an adequate gold standard. No information was available on sensitivity or specificity, and thus responsiveness scored poorly. Although* interpretability* is not considered a psychometric property, some comments can be made. No floor and ceiling effects were described. No Minimal Important Change (MIC) or Minimal Important Difference (MID) was calculated.

#### 3.3.2. SOAL [25]

*Internal consistency* scored poorly for similar reasons as the previous questionnaire: no factor analysis was performed and no other studies were available that provided this information, as Govender et al. [25] were the first to report on the SOAL.* Reliability* was not assessed. No SEM was calculated resulting in poor rating of* measurement error*. Again, as no reference to age, gender, disease characteristics, country, or setting was considered during item selection nor was any evaluation conducted to determine if all 17 items reflected the construct (dysphagia), and* content validity* received a poor rating. No information on* structural validity* was reported. The sample size was considered small (less than 30 subjects per analysis), thus resulting in a poor rating of* hypotheses testing*. When considering* criterion validity*, the authors used Pearson correlation coefficients instead of Spearman's Rho for correlations between ordinal data. Furthermore, it was not clear how missing responses to items were handled. Therefore, criterion validity scored fair.* Responsiveness* on the other hand scored poorly because no longitudinal design was used. As far as* interpretability* was concerned, no floor and ceiling effects, MIC, or MID were calculated.

#### 3.3.3. Self-Report Symptom Inventory/SSQ [26–29]

When determining* internal consistency*, Wallace et al. [26] used a moderate sample size but presented only a Pearson's product moment correlation matrix. No Cronbach's alphas were calculated. Thus internal consistency scored fair. In determining* reliability* and* measurement error*, small sample sizes were used (less than 30 per analysis). The percentage agreement was calculated but no weighted Kappa calculations were reported; SEM data were also missing. Both reliability and measurement error scored poorly.* Content validity* received a fair rating because the authors did not assess if all items were relevant to the purpose of the application of the questionnaire. In terms of* structural validity*, it was unclear how missing items were handled, resulting in a fair rating. Because no information was provided on the description of the constructs or measurement properties of the comparator instruments,* hypothesis testing* was considered poor. Information on* criterion validity* was not reported. In determining* responsiveness,* a moderated sample size (*N* = 45) was used, but no information was provided on how missing data were handled. Responsiveness was rated fair. Again, no floor and ceiling effects, MIC, or MID was reported (*interpretability*).

Dwivedi et al. [27] did not calculate* internal consistency*, but referred to another study in which factor analysis was performed, but not in a similar study population. Internal consistency was rated fair. The authors used a moderate sample size (*N* = 54) when determining* reliability*. Spearman correlation coefficients were calculated without providing evidence that no systematic change had occurred or with evidence that systematic change did occur. It was unclear whether the patients were stable.* Reliability* was considered to be fair. Measurement error was rated poorly as no SEM was calculated.* Content validity, structural validity,* or* hypotheses testing* was not assessed.* Criterion validity* was rated as fair, although it was unclear whether the criterion used could be considered an adequate “gold standard.”* Responsiveness* was not evaluated. Similar remarks as stated before regarding* interpretability*: no floor and ceiling effects, MIC, or MID was calculated.

Finally, two studies need to be mentioned briefly although their information on psychometric properties of the Self-report Symptom Inventory or SSQ is very limited. Dwivedi et al. [28] did not evaluate any psychometric properties, but calculated change scores (i.e., means and standard deviations) for relevant (sub)groups (e.g., for normative groups and subgroups of patients). Such information fits under* interpretability*, but again, no information on floor and ceiling effects, MIC, or MID was presented. Manjaly et al. [29] considered* responsiveness* using a small sample size (*N* = 9). No correlations were calculated nor did they use a criterion. Responsiveness was rated as poor. As in all previous studies, no floor and ceiling effects, MIC, and MID were calculated.

## 4. Discussion

When considering the restricted number of published FHS questionnaires available (Table 3) and the overall poor ratings on their psychometric properties (Table 7), it is evident that more research is needed in the area of FHS in oropharyngeal dysphagia. First, frequently authors did not evaluate all psychometric properties as defined by Mokkink et al. [11] resulting in missing data (“NR”). Secondly, when assessing the psychometric properties, authors seldom met the criteria as described in the 4-point rating scale [13]. Most studies simply failed to meet the “worse score counts” criteria. It seems that even though Terwee et al. [13] and Mokkink et al. [11] specialise in the evaluation of psychometric qualities of health-related questionnaires, their rating system appears to be so severe that it is unable to differentiate between the more subtle psychometric qualities of instruments. Although all the FHS questionnaires lacked sufficient validation, a need to distinguish between all the “poor” ratings seems desirable.

In general, most FHS questionnaires reported on in this study received poor overall methodological quality scores per measurement domain. However, when reevaluating the reliability and validity of these questionnaires according to preset quality criteria on psychometrics, it is possible that the methodological outcome per measurement property may show significant positive changes. If the overall methodological quality shows satisfactory improvement on most measurement properties, the use of the questionnaires in daily clinic and research can be justified. Conversely, without satisfactory improvement on measurement properties, new FHS questionnaires need to be developed using and reporting on preestablished psychometric criteria as recommended in the literature.

## 5. Conclusions

- A systematic literature search retrieved three original English-language FHS questionnaires: the Eating Assessment Tool (EAT-10) [24], the Swallowing Outcome After Laryngectomy (SOAL) [25], and the Self-report Symptom Inventory [26]. The Sydney Swallow Questionnaire (SSQ) [27] is identical to the previously published Self-report Symptom Inventory by Wallace et al. [26].
- The psychometric properties of all three FHS questionnaires were determined using the COSMIN taxonomy of measurement properties and definitions for health-related patient-reported outcomes [11], the COSMIN checklist [12], and the psychometric criteria using a 4-point rating scale according to Terwee et al. [13]; all three FHS questionnaires obtained poor overall methodological quality scores for most psychometric properties.
- All FHS questionnaires need psychometric reassessment; if the overall methodological quality shows satisfactory improvement on most measurement domains, the use of the questionnaires in daily clinic and research can be justified. However, in cases of insufficient validity and/or reliability scores, it is recommended to develop new FHS questionnaires using and reporting on preestablished psychometric criteria as suggested in literature.
- In general when assessing the validity and reliability of FHS or health-related questionnaires, researchers must use preestablished quality criteria like Terwee et al. [13] when reporting on psychometric properties of their instrument.
- Arguably the most important conclusion may be that academics should be educated on the psychometric domains that require reporting when developing and validating a FHS questionnaire or any other health-related questionnaire.

## Figures and Tables

**Table 1:** Functional health status questionnaires in oropharyngeal dysphagia: search strategy.

	Literature database	Search terms	Limits	Number of abstracts identified (*N*_total_ = 2703)
Mesh or thesaurus terms	Pubmed	(“Questionnaires”[Mesh] or “Health Status”[Mesh] or “Severity of Illness Index”[Mesh:NoExp]) and (“deglutition disorders”[Mesh:NoExp] or “Deglutition”[Mesh])	N.A.	1218
	Embase	(exp QUESTIONNAIRE/) and (exp DYSPHAGIA/or exp swallowing)	N.A.	1196

Free text words^1^ (truncation or wild card)	Pubmed	(questionnaire*[all fields] or “functional health status"[all fields]) and (“deglutition”[All Fields] or “deglutition disorders"[All Fields] or “deglutition disorder“[All Fields] or (“oropharyngeal"[All Fields] and “dysphagia"[All Fields]) or “oropharyngeal dysphagia"[All Fields] or swallow* or ((dysphag*) and (oropharynx or oropharyng*)))	From 5-6-2012 to 5-6-2013	195
	Embase	(questionnaire*[all fields] or “functional health status"[all fields]) and (“deglutition"[All Fields] or “deglutition disorders"[All Fields] or “deglutition disorder"[All Fields] or (“oropharyngeal"[All Fields] and “dysphagia"[All Fields]) or “oropharyngeal dysphagia"[All Fields] or swallow* or ((dysphag*) and (oropharynx or oropharyng*)))	From 2012 to current	94

^1^Similar free text words were used for both Pubmed and Endbase.

**Table 2:** COSMIN: definitions of psychometric domains and properties for Health-Related Patient-Reported Outcomes (HR-PRO).

Domain	Psychometric property	Aspect of a psychometric property	Definition^1^
Reliability			The degree to which the measurement is free from measurement error
	Internal consistency		The degree of the interrelatedness among the items
	Reliability		The proportion of the total variance in the measurements which is because of “true” differences among patients
	Measurement error		The systematic and random error of a patient's score that is not attributed to true changes in the construct to be measured

Validity			The degree to which an HR-PRO instrument measures the construct(s) it purports to measure
	Content validity		The degree to which the content of an HR-PRO instrument is an adequate reflection of the construct to be measured
		Face validity	The degree to which (the items of) an HR-PRO instrument indeed looks as though it is an adequate reflection of the construct to be measured
	Construct validity		The degree to which the scores of an HR-PRO instrument are consistent with hypotheses based on the assumption that the HR-PRO instrument validly measures the construct to be measured
		Structural validity	The degree to which the scores of an HR-PRO instrument are an adequate reflection of the dimensionality of the construct to be measured
		Hypotheses testing	Idem construct validity
		Cross-cultural validity	The degree to which the performance of the items on a translated or culturally adapted HR-PRO instrument is an adequate reflection of the performance of the items of the original version of the HR-PRO instrument
	Criterion validity		The degree to which the scores of an HR-PRO instrument are an adequate reflection of a “gold standard”

Responsiveness	Responsiveness		The ability of an HR-PRO instrument to detect change over time in the construct to be measured

Interpretability^2^			The degree to which one can assign qualitative meaning to an instrument's quantitative scores or change in scores.

^1^Definitions derived from Mokkink et al. [11].

^2^Interpretability is not considered a psychometric property [11].

**Table 3:** Overview of functional health status questionnaires: reasons for inclusion and exclusion.

	Questionnaire	Acronym	Inclusion	Exclusion
1	Eating Assessment Tool [24]	EAT-10	Mainly oropharyngeal dysphagia related FHS	

2	Swallowing Outcome After Laryngectomy [25]	SOAL	Mainly oropharyngeal dysphagia related FHS	

3	Self-report Symptom Inventory [26]	N/A	Mainly oropharyngeal dysphagia related FHS	
	Sydney Swallow Questionnaire^1^ [27]	SSQ		

4	Caregiver mealtime and Dysphagia Questionnaire [23]	CMDQ		Target population: care givers

5	Dysphagia Disorders Survey [22]	DDS		Questionnaire for speech pathologists during patient's mealtime observation; target population: residential populations with intellectual disabilities

6	Deglutition Handicap Index [17]	DHI		Combination of oropharyngeal dysphagia related HR-QoL and FHS; French language

7	Dysphagia Handicap Index [15]	DHI		Combination of oropharyngeal dysphagia related HR-QoL and FHS;

8	Dysphagia Short Questionnaire [18]	DSQ	(Mainly oropharyngeal dysphagia related FHS)	Swedish language

9	Dysphagia in Multiple Sclerosis questionnaire [19]	DYMUS	(Mainly oropharyngeal dysphagia related FHS)	Italian language

10	Mayo Dysphagia Questionnaire-30 [21]	MDQ-30	(Mainly oropharyngeal dysphagia related FHS)	Target population^2^: reflux esophagitis and/or reflux peptic stricture

11	MD Anderson Dysphagia Inventory [16]	MDADI		Mainly oropharyngeal dysphagia related HR-QoL

12	Swallowing Disturbance Questionnaire [20]	SDQ	(Mainly oropharyngeal dysphagia related FHS)	Hebrew language

^1^SSQ is identical to the modified Self-report Symptom Inventory [26].

^2^Information provided by corresponding author.

**Table 4:** Description of studies for the *development* of questionnaires for the assessment of FHS in oropharyngeal dysphagia.

Questionnaire	Reference	Target population	Study design	Study population	Age in years (mean/median, SD, and range)^2^
EAT-10	Belafsky et al., 2008 [24]	Orophageal and esophageal dysphagia	Cross-sectional study	Development EAT-20 (I) Multidisciplinary group of dysphagia experts (gastroenterologists, otolaryngologists, SLTs, nutritionists). Development EAT-10 (II) Healthy subjects (*N* = 100); (III) Subjects with voice and swallowing disorders (*N* = 235; 127 M, 108 F); Diagnosis: reflux disease (*N* = 66; 28%), voice disorder (*N* = 51; 22%), oropharyngeal dysphagia (including stroke, progressive neurologic disease, ALS, or pseudobulbar palsy; *N* = 50; 21%), head and neck cancer (*N* = 42; 18%), esophageal dysphagia (including esophageal motility disorders, neoplasia, webs, strictures, or rings; *N* = 26; 11%).	(II) NR (III) Mean = 62 (SD = 14); range = NR

SOAL	Govender et al., 2012 [25]	After total laryngectomy	Cross-sectional study	(I) Volunteer laryngectomees from all regions of the UK; mean of 5 years after surgery (*N* = 10). (II) Experienced SLTs (*N* = 6): item generation. (III) Focus groups of SLPs (*N* = 35): content consensus.	(I) Mean = 63; range = NR

Self-report Symptom Inventory^1^	Wallace et al., 2000 [26]	Oropharyngeal dysphagia	Cross-sectional study	Development Prototype Self-report Inventory (19 items): clinical judgement by one experienced clinician (senior author) and one SLP. Development Modified Self-report Inventory (17 items): (I) oropharyngeal dysphagic patients with neuromyogenic etiology (*N* = 45; 27 M, 18 F); diagnosis: CVA—brainstem (*N* = 12), CVA—other (*N* = 2), Parkinson disease (*N * = 12), Movement disorder—other (*N* = 4), Myopathy (*N* = 3), MG (*N* = 1), ALS (*N* = 6), brainstem lesion—other (*N* = 2), idiopathic (*N* = 3).	(I) Mean = 70 (SD = 13); range = 30–96

^1^The *Modified* Self-report Symptom Inventory (17 items) is based on the *Prototype* Self-report Symptom Inventory (19 items) by Wallace et al. [26]. The Sydney Swallow Questionnaire (SSQ) named by Dwivedi et al. [27] is identical to the previously published Modified Self-report Symptom Inventory by Wallace et al. [26].

^2^NR: not reported.

**Table 5:** Description of *validation* studies related to questionnaires for the assessment of FHS in oropharyngeal dysphagia.

Questionnaire	Reference	Target population	Study design	Study population	Age in years (mean/median, SD, and range)^1^
EAT-10	Belafsky et al., 2008 [24]	Orophageal and esophageal dysphagia.	(I) and (II) Cross-sectional study and (III) pre- and postintervention measurement	(I) Community cohort of healthy subjects (with no medical history of voice, swallowing, reflux, airway, neurologic, rheumatologic, hematologic, or neoplastic disorders). Centre for voice and swallowing: (II) Subjects with voice and swallowing disorders; diagnosis: reflux disease (*N* = 66; 28%), voice disorder (*N* = 51; 22%), oropharyngeal dysphagia (including, stroke, progressive neurologic disease, ALS, or pseudobulbar palsy; *N* = 50; 21%), head and neck cancer (*N* = 42; 18%), esophageal dysphagia (including esophageal motility disorders, neoplasia, webs, strictures, or rings; *N* = 26; 11%). (III) Patients with Zenker's diverticulum (*N* = 23; 50%), reflux disease (*N* = 14; 30%), esophageal stricture (*N* = 9; 20%).	(I) Healthy subject (*N* = 100; 53 M, 47 F); mean age = 48 (SD = 16); range = NR. (II) Subjects with voice and swallowing disorders (*N* = 235; 127 M, 108 F); mean = 62 (SD = 14); range = NR. (III) Patients undergoing treatment for dysphagia (*N* = 46; 21 M, 25 F); mean age = 65 (SD = 16); range = NR

SOAL	Govender et al., 2012 [25]	After total laryngectomy	Cross-sectional study	Large urban teaching hospital	(I) Laryngectomees: experimental group (*N* = 19; 10 M, 9 F); mean age = 66 (range = 48–80). (II) Noncomplaining volunteers: controls (*N* = 20; 10 M, 10 F); mean age = 63 (range = 44–83) (III) ≥3 months after head and neck chemoradiotherapy with known dysphagia (*N* = 19); mean age = 61 (range = 41–92)

Self-report Symptom Inventory	Wallace et al., 2000 [26]	Oropharyngeal dysphagia	(I), (II), and (III) Cross-sectional study and (II) pre- and postintervention measurement	(I) Oropharyngeal dysphagic patients with neuromyogenic etiology (*N* = 45; 27 = M, 18 F); diagnosis: CVA—brainstem (*N* = 12), CVA—other (*N* = 2), Parkinson disease (*N* = 12), Movement disorder—other (*N* = 4), Myopathy (*N* = 3), MG (*N* = 1), ALS (*N* = 6), brainstem lesion—other (*N* = 2), idiopathic (*N* = 3). (II) Oropharyngeal dysphagic patients as a result of Zenker's diverticulum (*N* = 11; 6 M, 5 F). (III) Nondysphagic controls: urology patients, gynaecology patients (*N* = 19; 8 M, 11 F).	(I) Mean = 70 (SD = 13); range = 30–96 (II) Mean = 66 (SD = 13); range = 36–84 (III) Mean = 62 (SD = 16); range = 31–94

(SSQ)	Dwivedi et al., 2010, Dwivedi et al., 2012 [27, 28]	Oral and oropharyngeal cancer	Cross-sectional study	After oral or oropharyngeal cancer (*N* = 54; 35 M, 19 F).	Mean = 58.6 (SD = 9.7); range = 35.9–80

(SSQ)	Manjaly et al., 2012 [29]	Oculopharyngeal muscular dystrophy (OPMD)	Before and after repeat intervention measurement	Repeat cricopharyngeal dilatation in OPMD (*N* = 9; 2 M, 7 F).	Mean = 60.1 (9.0); range = 50–77

^1^NR: not reported.

**Table 6:** Characteristics of questionnaires for the assessment of FHS in oropharyngeal dysphagia.

Questionnaire	Domains (number of items)	Scales	Number of items	Response options	Range of scores^1^	Cut-off point^2^
EAT-10	Severity of oropharyngeal dysphagia	1	10	5-point Likert scale: 0 = no problem, 4 = severe problem	0–40	0–2 is in normal range; 3–40 is indicative of swallowing problems

SOAL	Swallowing function	1	17	(i) No, a little, a lot (ii) (If answered “a little” or “a lot”: Does this bother you? Yes/No)	0–34	0–4 is within normal range; 5–34 is indicative of swallowing problems

Self-report Symptom Inventory	Symptomatic severity of oropharyngeal dysphagia	1	19 *Prototype* Self-report Symptom Inventory	(i) 17 items: visual analogue scales anchored by extreme statements (normal function—extreme dysfunction) (ii) 1 item: 0 to 5 (iii) 1 item: 0 to 3	0–1708	0–192 is within normal range; 193–1708 is indicative of swallowing problems
			17 *Modified* Self-report Symptom Inventory	(i) 16 items: visual analogue scales anchored by extreme statements (normal function—extreme dysfunction) (ii) 1 item: 0 to 5	0–1700	

(SSQ)	Overall swallowing function (1); direct physiological swallowing function (15); swallowing-related QoL (1)	1	170	Visual analogue scales anchored by extreme statements (normal function—extreme dysfunction)	0–1700	NR^3^

^1^Higher scores indicate a more severe swallowing impairment.

^2^EAT-10 cut-off score: based on normative data using the mean total symptom score plus two SD (i.e., 0.40 + [2 × 1.01] ≥ 2.42) as upper limit of normal in healthy subjects (*N* = 100); SOAL cut-off score: based on regression analysis of Modified Barium Swallow and SOAL consensus rating determined a score of 5 and greater on the SOAL to be indicative of OD problems; Self-report Symptom Inventory (SSQ) cut-off score: based on normative data using the mean total score plus two SD (i.e., 67 + [2 × 63] ≥ 193) as upper limit of normal in healthy subjects (*N* = 19).

^3^NR = not reported.

**Table 7:** Overview of the psychometric properties of FHS questionnaires in oropharyngeal dysphagia [11–13].

Questionnaire	Measurement property^2^							
	Internal consistency	Reliability	Measurement error	Content validity	Structural validity	Hypotheses testing	Criterion validity	Responsiveness
EAT-10	Poor	Poor	Poor	Poor	NR	Poor	NR	Poor
SOAL	Poor	NR	Poor	Poor	NR	Poor	Fair	Poor
Self-report Symptom Inventory/SSQ^1^	Fair	Poor/fair	Poor	Fair	Fair	Poor	Fair	Fair

^1^The SSQ [27] is identical to the Self-report Symptom Inventory [26].

^2^NR: not reported.
